# Selective Pressure Promotes Tetracycline Resistance of *Chlamydia Suis* in Fattening Pigs

**DOI:** 10.1371/journal.pone.0166917

**Published:** 2016-11-28

**Authors:** Sabrina Wanninger, Manuela Donati, Antonietta Di Francesco, Michael Hässig, Karolin Hoffmann, Helena M. B. Seth-Smith, Hanna Marti, Nicole Borel

**Affiliations:** 1 Institute for Veterinary Pathology, University of Zurich, Vetsuisse Faculty, Zurich, Switzerland; 2 DIMES, Microbiology, Policlinico S. Orsola, University of Bologna, Bologna, Italy; 3 Department of Veterinary Medical Sciences, University of Bologna, Bologna, Italy; 4 Department for Farm Animals, University of Zurich, Vetsuisse Faculty, Zurich, Switzerland; Veterinary Pathology, SWITZERLAND

## Abstract

In pigs, *Chlamydia suis* has been associated with respiratory disease, diarrhea and conjunctivitis, but there is a high rate of inapparent *C*. *suis* infection found in the gastrointestinal tract of pigs. Tetracycline resistance in *C*. *suis* has been described in the USA, Italy, Switzerland, Belgium, Cyprus and Israel. Tetracyclines are commonly used in pig production due to their broad-spectrum activity and relatively low cost. The aim of this study was to isolate clinical *C*. *suis* samples in cell culture and to evaluate their antibiotic susceptibility *in vitro* under consideration of antibiotic treatment on herd level.

Swab samples (n = 158) identified as *C*. *suis* originating from 24 farms were further processed for isolation, which was successful in 71% of attempts with a significantly higher success rate from fecal swabs compared to conjunctival swabs. The farms were divided into three treatment groups: A) farms without antibiotic treatment, B) farms with prophylactic oral antibiotic treatment of the whole herd consisting of trimethoprime, sulfadimidin and sulfathiazole (TSS), or C) farms giving herd treatment with chlortetracycline with or without tylosin and sulfadimidin (CTS). 59 isolates and their corresponding clinical samples were selected and tested for the presence or absence of the tetracycline resistance class C gene [*tet*(C)] by conventional PCR and isolates were further investigated for their antibiotic susceptibility *in vitro*. The phenotype of the investigated isolates was either classified as tetracycline sensitive (Minimum inhibitory concentration [MIC] < 2 μg/ml), intermediate (2 μg/ml ≤ MIC < 4 μg/ml) or resistant (MIC ≥ 4 μg/ml). Results of groups and individual pigs were correlated with antibiotic treatment and time of sampling (beginning/end of the fattening period). We found clear evidence for selective pressure as absence of antibiotics led to isolation of only tetracycline sensitive or intermediate strains whereas tetracycline treatment resulted in a greater number of tetracycline resistant isolates.

## Introduction

Since the 1940s, antibiotics have been prescribed in human and veterinary medicine to treat bacterial infection, but only a few years after discovery of these antimicrobial drugs, the first cases of acquired antibiotic resistance emerged in the form of penicillin-resistant *Staphylococcus aureus* [1]. Antibiotic resistance as a result of chromosomal mutation or acquisition of resistance genes is promoted by numerous factors including a) the use of sub-inhibitory antimicrobial concentrations (during treatment, as preventive measures or as growth promoters in livestock), b) the use of broad-spectrum antibiotics, and c) non-compliance of individuals and communities under treatment. Moreover, there is a positive correlation between the frequency of antibiotic treatment and the occurrence of resistance [2]. Taken together, the use of antibiotics exerts selective pressure against the microbial community promoting the emergence of therapy-resistant bacteria [3]. However, selective pressure does not only concern pathogens. Complex microbial ecosystems, in particular the microbiota of the gastrointestinal tract, have been reported to regularly acquire and transfer antibiotic resistance genes, often promoted by the use of oral antimicrobial drugs. With high bacterial loads of 10^11^ to 10^12^ bacteria/ml from several phyla, the colon offers plenty of opportunity for horizontal gene transfer and the selection for commensal bacteria resistant to antibiotics [4, 5].

Of particular interest in this wide range of commensal and opportunistic bacteria is the species *Chlamydia suis*, which is primarily found in the gastrointestinal tract of pigs and was the first obligate intracellular bacterium reported to have acquired stable tetracycline resistance through horizontal gene transfer [6, 7]. *C*. *suis* belongs to the *Chlamydiaceae*, a family of Gram-negative bacteria which also includes *C*. *trachomatis*, the leading cause of sexually transmitted bacterial disease worldwide as well as the causing agent of blinding trachoma [8]. Despite its high prevalence in pigs, with up to 94.2% in farms, *C*. *suis* is not considered a primary pathogen for pigs, but it has been associated with several disease complexes including conjunctivitis as well as reproductive disorders, and cases of diarrhea within the herd related to a high *C*. *suis* prevalence [9, 10]. The tetracycline resistance found in *C*. *suis* is defined by the presence of an efflux pump encoding gene called tetracycline resistance gene class C [*tet*(C)] within a genomic island [7], though its presence does not necessarily translate into antibiotic resistance *in vitro* [11]. *C*. *suis* strains carrying the *tet*(C) gene have been identified in several pig farms in the USA, Italy, Switzerland, Belgium, Cyprus and Israel in recent years [7, 12–14, 15]. Moreover, there has been preliminary evidence for selective pressure as a result of antibiotic treatment in *C*. *suis*-infected pigs [13].

In this study, we used 158 swab samples from a vast collection of over 2000 fecal and conjunctival swabs originating from 29 farms [9]. We found that 1) the estimated number of gene copies in a clinical sample by real-time PCR is a good predictor to assess successful isolation, and 2) isolation from conjunctival swabs is far less successful compared to fecal samples suggesting that fecal contamination rather than actual conjunctival infection occurred. A selection of isolates (n = 59) were further tested for *tet*(C) by PCR as well as their antibiotic susceptibility *in vitro*, confirming previous findings that positive *tet*(C) results do not necessarily mean that the isolate is also phenotypically resistant [11]. Furthermore, we show that the number of tetracycline resistant *C*. *suis* isolates in pigs treated with tetracycline derivatives tends to increase between the beginning and end of the fattening period, whereas farms where no antibiotic treatment was applied only yielded tetracycline sensitive or intermediate *C*. *suis* isolates, providing evidence for selective pressure.

## Material and Methods

### Sample collection and study design

Between December 2014 and September 2015, samples were collected from 636 pigs in 29 farms in the central part of Switzerland. Each pig was sampled at the beginning (first sampling) and end (second sampling) of the fattening period (total fattening period of around 3 months). Two conjunctival (both eyes, pooled) and two fecal swabs (FLOQSwabs^®^, Copan Italia, Brescia, Italy) were collected per sampling (two timepoints), of which one swab per anatomical site was used for DNA extraction and the other was stored at—80°C in sucrose phosphate transport medium, resulting in a total of eight flocked swabs per pig [9].

**Fig 1:**
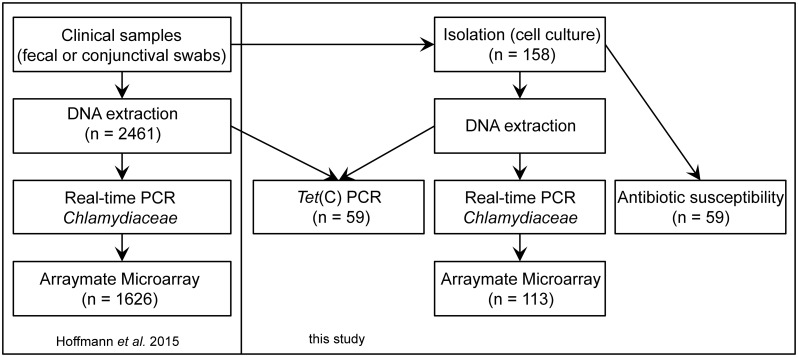
Study Design. Shown is the study design as well as the sample numbers used for each method applied in this study and that of Hoffmann et al. [9].

In the present study 158 swab samples [9], comprising 21 conjunctival and 137 fecal swabs belonging to 24 farms, were further processed for isolation. The farms were divided into three groups: A) farms without antibiotic treatment (n = 16) and B) farms prophylactically treating the whole herd with trimethoprime, sulfadimidin and sulfathiazole (TSS, n = 3), or C) chlortetracycline with or without tylosin and sulfadimidin (CTS, n = 5) (S1 Table). A selection of isolates (n = 59) and their corresponding clinical samples were tested for the presence or absence of the tetracycline resistance class C gene [*tet*(C)] by conventional PCR and were further investigated for their antibiotic susceptibility, as described in detail below (Fig 1).

### Host cells and media

The isolation of swab samples and antibiotic susceptibility assays were performed in LLC-MK2 cells (continuous Rhesus monkey kidney cell line, provided by IZSLER Brescia, Italy) grown on glass coverslips (Ø 12 mm) in 7 ml Trac bottles (Thermo Fisher Scientific, Waltham, MA, USA) at 37°C and 5% CO_2_.

Cells were maintained in growth medium containing 500 ml Eagle’s minimum essential medium (EMEM, Gibco, Thermo Fisher Scientific, Invitrogen, Carlsbad, CA, USA) supplemented with 10% heat-inactivated fetal calf serum (FCS, BioConcept, Allschwil, Switzerland), 0.6 ml fungizone (250 μg/ml) (Gibco), 0.1 ml gentamycin (50 mg/ml) (Gibco), 0.5 ml vancomycin (10 mg/ml) (Gibco) and 6 ml glucose (0.06 g/ml) (Sigma-Aldrich Co., St. Louis, MO, USA). Following infection, growth medium was replaced by chlamydiae cultivation medium consisting of 500 ml EMEM supplemented with 20% FCS (BioConcept), 5 ml L-glutamine (Gibco, Thermo Fisher Scientific), 4 ml fungizone (250 μg/ml), 0.5 ml gentamycin (50 mg/ml), 5 ml vancomycin (10 mg/ml), 2 g D-(+)-glucose (Sigma-Aldrich) and 0.7 ml cycloheximide (1 mg/ml) (Sigma-Aldrich) as described by Donati et al. [16].

Clinical samples for isolation were frozen and stored in sucrose phosphate (SP) transport medium consisting of 37.5 g sucrose, 0.2 M Na_2_HPO_4_-2H_2_O and 0.2 M Na_2_PO_4_-2H_2_O in 500 ml ddH2O. Isolates for the antibiotic susceptibility assay were stored in SP supplemented with 160 μl fungizone (final concentration: 2 μg/ml), 80 μl gentamycin (200 μg/ml) and 40 μl vancomycin (100 μg/ml).

### Isolation of *Chlamydia*

Fecal (n = 137) and conjunctival (n = 21) swabs, originally sampled in 400 μl SP medium and stored at– 80°C, were chosen for isolation. Isolates obtained from the pigs in this study were the same samples that were collected previously as described by Hoffmann et al. [9].

Specifically, at a cell confluence of 100%, swab samples were thawed, vortexed for 1 min and inoculated onto LLC-MK2 cells in triplicate (~ 133.3 μL) together with chlamydiae cultivation medium (final volume per Trac bottle: 2 ml) before centrifugation for 2 hours at 2385 g (33°C). After an incubation period of 48 h (37°C, 5% CO_2_), one Trac bottle from each sample was fixed in methanol for 10 min before detection of intracellular chlamydial inclusions by immunofluorescence assay (IFA) with a DMLB fluorescence microscope (Leica Microsystems, Wetzlar, Germany). Briefly, monolayers were air-dried and placed onto a paraffin-embedded plate mounted with 5 μL fluorescein-conjugated monoclonal antibody specific for the chlamydial LPS genus-specific antigen (IMAGEN Chlamydia K610111-2, Thermo Fisher Scientific). After 30 min incubation at 37°C, coverslips were washed three times in PBS and fixed onto glass slides using FluoreGuard Mounting (Hard Set, ScyTek Laboratories Inc., Logan, UT, USA).

For monolayers with visible inclusions, the remaining Trac bottles of the corresponding sample were passaged by vortexing and inoculation of new monolayers until the rate of chlamydial infection achieved 80–100%. Successfully isolated strains were then frozen in SP medium with antibiotics (gentamycin, vancomycin and fungizone), heat-treated FCS or sterile PBS and stored at—80°C or—20°C for further investigation.

Cultures were considered negative for viable *Chlamydia* if no inclusions were detected after three passages.

### Confirmation of chlamydial species

#### DNA extraction and real-time PCR for *Chlamydiaceae*

DNA of isolated stocks was extracted using the QIAamp DNA mini kit (Qiagen, Hilden, Germany), following the supplier’s recommendations. All samples were examined using real-time PCR based on *Chlamydiaceae* family-specific 23S rRNA gene primers performed on an ABI 7500 instrument, as previously described [17, 18].

All samples were tested in duplicate and the cycle threshold was set at 0.1 for each run. A mean cycle threshold (Ct value) < 38 was considered positive, and was used to calculate the corresponding chlamydial load as *Chlamydiaceae* 23S rRNA gene copy number per μl. If the amplification of internal control DNA was inhibited, the run was repeated following 1:10 dilution of the sample. The positive control contained a sevenfold dilution series of *C*. *abortus* DNA and a negative control of water instead of the template DNA was included in each run [9].

#### Arraymate Microarray for species identification

All samples with positive results in the real-time PCR were further investigated using a species-specific 23S rRNA Arraymate microarray assay (Alere, Jena, Germany), as established by [19]. The current version carries 34 probes for eleven *Chlamydiaceae* species, three genus-specific probes, four family markers and 15 probes for *Chlamydia*–like organisms. Additionally, there are four internal control DNA probes and an internal staining control (biotin marker) [9]. Each sample, including internal control DNA (Intype IC-DNA, Qiagen Labor, Leipzig, Germany), was amplified and biotin-labeled using a biotinylation PCR, as described by Borel et al. [19], with 10 min of initialization (96°C) and 40 cycles of 94°C (denaturation), 50°C (annealing), and 72°C (elongation) for 30 s each. 2–4 μl of amplification product was loaded on the chip, which was processed according to manufacturer’s instructions.

#### *Tet*(C) PCR

DNA of 59 isolates and their corresponding clinical samples were screened with an established PCR amplifying a 525 bp fragment of the *tet*(C) gene according to Dugan et al. [7]: CS43 (5’-AGCACTGTCCGACCGCTTTG-3’) and CS47 (5’-TCCTCGCCGAAAATGACCC-3’). 5 μl DNA per sample was added to a PCR mixture (final volume 50 μl) containing 1.5 U AmpliTaq Gold DNA polymerase (Applied Biosystems/Roche), 2.0 mM magnesium chloride, 200 μM of each deoxynucleoside triphosphate, 1x Gold Buffer and 20 pmol of each primer. Cycling conditions consisted of initial denaturation (4 min, 94°C) followed by 40 cycles of denaturation (94°C), annealing (55°C) and chain elongation (72°C) for 1 min each. The reaction was completed with a final elongation step (72°C) for 7 min.

### Antibiotic susceptibility

Titration by sub-passage was performed on all the isolates (n = 59, stored in SP medium). Briefly, chlamydiae-containing SP media were sonicated for 5 min (Brandson 250 Sonifier, Danbury, CT, USA), inoculated onto 100% confluent LLC-MK2 cells in Trac bottles at a 10-fold dilution and centrifuged at 2385 g (33°C) for 1 h. After 48 h incubation at 37°C in the presence of 5% CO_2_, cultures were fixed and immunolabeled. For analysis, the number of inclusions at 200-fold magnification throughout the entire coverslip was counted and used to determine the number of inclusion-forming units per milliliter (IFU/ml). Each antibiotic susceptibility determination was performed with approximately 5 x 10^3^ IFU/ml per investigated isolate as previously described [11].

Following inoculation of 16 confluent monolayers, Trac bottles were centrifuged at 2385 g (33°C) for 1 hour, inocula were removed and replaced with chlamydiae cultivation medium containing a serial two-fold dilution of tetracycline (Sigma-Aldrich) at concentrations ranging from 0.06 μg/ml to 4 μg/ml. Each tetracycline concentration was added in duplicate. Following incubation at 37°C for 48 h, one infected monolayer per tetracycline concentration was fixed with methanol, and immunolabeled, as described above (Material and Methods section). The minimum inhibitory concentration (MIC) of tetracycline was defined as “the lowest concentration preventing the detection of more than 90% of the chlamydial inclusions compared with the drug-free control” [11, 16]. In parallel, media of the remaining Trac bottles was replaced with tetracycline-free chlamydiae cultivation medium after washing the coverslips with PBS. Monolayers were fixed after 48 h of incubation and immunolabeled for the evaluation of the minimum bactericidal concentration (MBC), which was identical to MIC determination. *C*. *suis* isolates with a MIC/MBC of ≥ 4 μg/ml were defined as resistant, whereas cultures with 2 μg/ml ≤ MIC/MBC < 4 μg/ml were considered intermediate and isolates with a MIC/MBC of < 2 μg/ml were sensitive.

### Statistical analysis

The online calculator N-1 Two Proportion test (http://www.measuringu.com/ab-calc.php) was used to compare the isolation success regarding swab origin (rectal or conjunctival swab) and the chlamydial load. The calculator is based on a two-tailed chi-square test. A p-value < 0.05 was considered significant.

## Results

### High bacterial loads result in more successful isolation

Isolation was considered successful if chlamydial inclusions were detected after 1 to 3 passages by immunofluorescence assay. Subsequently, species confirmation was performed by real-time PCR and Arraymate microarray. In this study, 113 of the 158 *Chlamydia* positive swabs produced *Chlamydia*-positive cultures, resulting in a success rate of 71.5% (Table 1). Interestingly, there was a significant difference between the success of the conjunctival swabs (p-value < 0.05), with only one isolate out of 21 samples (4.8%) and fecal swabs where the success rate was 82.5%.

**Table 1:** Number of successful isolations and success rates depending on swab origin.

	Successful Isolations	All Samples	Success Rate [%]
Conjunctival Swabs	1	21	4.8
Fecal Swabs	112	137	82.5
**All Swabs**	**113**	**158**	**71.5**

**Fig 2:**
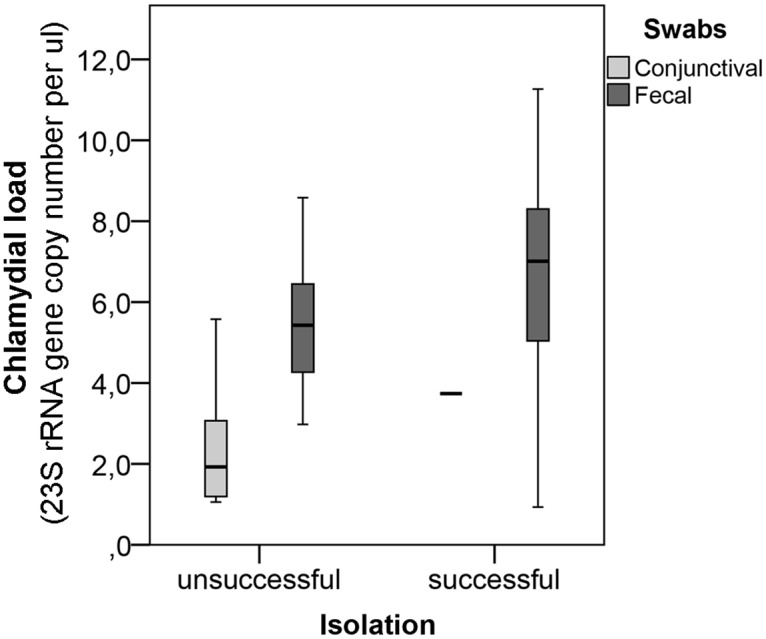
Comparison between the bacterial load of successful and failed isolations, depending on the anatomical site of sampling. Bacterial load of the clinical samples used for isolation expressed as 23S rRNA gene copy numbers per μl in a logarithmic scale (ln). The bacterial load of successfully isolated clinical swabs is compared to unsuccessful isolations while fecal and conjunctival swabs are presented separately.

We then proceeded to investigate these results by using the estimated chlamydial load from each clinical swab, as determined by real-time PCR to compare successful with unsuccessful isolations [9]. We further differentiated between fecal and conjunctival swabs. The distribution of copy numbers per sample was expressed by means of a boxplot, which clearly demonstrates that the general bacterial load of conjunctival swabs was lower compared to fecal swabs (Fig 2). Since the copy numbers per μl depend on the number of cycles in the *Chlamydiaceae* real-time PCR, they were expressed in a logarithmic scale leading to the exclusion of samples with mean copy numbers of less than 1 per μl (n = 15) for this analysis. Also excluded from this analysis were samples used for isolation where DNA of corresponding clinical swabs had to be diluted 10-fold in order to successfully perform the real-time PCR due to high bacterial loads (n = 13).

Fig 2 also indicates that the bacterial load of successful isolations was slightly higher compared to unsuccessful isolations. We decided to investigate this hypothesis by classifying the bacterial load of samples into four different groups: a) less than (<) 1, b) 1 to 100, c) 100 to 700 and d) more than (>) 700 copy numbers per μl, as shown in Table 2. For this analysis, we included all samples used for isolation by making the assumption that the 13 samples, of which a 10-fold dilution had to be performed, yielded more than 700 DNA copy numbers per μl. Eight of the diluted samples even yielded more than 700 copies, while four generated between 100 and 700 copies per μl, and one had only 39.7 copies per μl (S1 Table). The isolations from these samples were altogether successful.

**Table 2:** Successful isolation in relation to the copy numbers per μl of clinical swab samples.

Copy numbers per μl	< 1	1–100	100–700	> 700
Successful Isolation (n = 113)	4	18	28	63
Unsuccessful Isolation (n = 45)	11	20	11	3
**Success Rate [%]**	**26.7**	**47.4**	**71.8**	**95.5**

Using this classification, we were able to show that the isolation success correlated with the bacterial load. In detail, while less than 50% of all isolation attempts were successful if the corresponding clinical swabs yielded between 1 and 100 copy numbers per μl, the success rate of swabs yielding over 700 copies per μl was 95.5% (Table 2, S1 Table). The isolation success of swabs with over 700 copies per μl was significantly higher than that of swabs with fewer than 700 copies per μl (p < 0.05).

### *Tet*(C) PCR does not correlate with the *in vitro* phenotype

A recent publication has demonstrated that positive PCR results do not necessarily translate into a tetracycline resistant phenotype by means of an antibiotic susceptibility assay [11]. In view of these findings, we selected 59 *C*. *suis* isolates, analyzed their tetracycline susceptibility *in vitro* and subsequently compared them to the presence or absence of the *tet*(C) gene by PCR.

An isolate was considered *tet*(C)-positive if a band was present on the gel at 525 base pairs (bp) following amplification of the gene by PCR (S1 Fig). In total, 32 isolates were positive for *tet*(C), while 27 were negative. Regarding the antibiotic susceptibility assay, both the MIC and MBC were evaluated. The phenotype of investigated isolates was either classified as tetracycline sensitive (MIC < 2 μg/ml), or intermediate (2 μg/ml ≤ MIC < 4 μg/ml) or resistant (MIC ≥ 4 μg/ml), depending on the MIC concentration. In total, 31 out of the 59 investigated isolates were phenotypically sensitive (52.5%), six were considered intermediate (10.2%), while 22 were tetracycline resistant (37.3%) by the antibiotic susceptibility assay. Compared to MIC, the MBC values were generally higher by 0 to 0.5 μg/ml and 1.5 μg/ml in one case (S2 Table).

**Fig 3:**
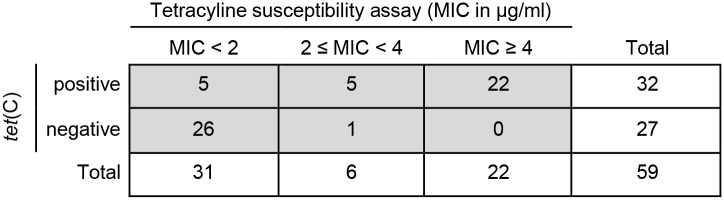
Tetracycline susceptibility assay (MIC in μg/ml) compared to *tet*(C) PCR. Shown are the *tet*(C) results by PCR (positive or negative) in the y-axis compared to the tetracycline susceptibility *in vitro* (MIC in μg/ml) in the x-axis for each individual isolate (n = 59). The *in vitro* phenotype is divided into three groups: sensitive (MIC < 2 μg/ml), intermediate (2 ≤ MIC < 4 μg/ml) and resistant (MIC ≥ 4 μg/ml).

Interestingly, while all phenotypically resistant isolates were also positive by the *tet*(C) PCR (22 / 22) as expected, sensitive or intermediate phenotypes could be both *tet*(C)-positive or negative. In fact, while 26 out of 31 phenotypically sensitive isolates tested *tet*(C)-negative, only one intermediate isolate was negative for *tet*(C) (Fig 3).

**Fig 4:**
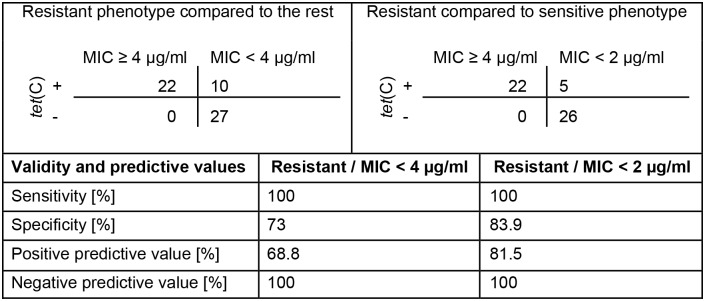
Validity and predictive values of the *tet*(C) PCR compared to *in vitro* susceptibility. Shown are the validity (sensitivity, specificity) and the predictive values of the *tet*(C) PCR compared to the phenotype by antibiotic susceptibility assay *in vitro*. The upper left panel and the middle column show the comparison between the resistant phenotype (MIC ≥ 4 μg/ml) and the sensitive or intermediate phenotype (MIC < 4 μg/ml). The upper right panel and the right column compare the resistant with only the sensitive phenotype (MIC < 2 μg/ml).

Since the antibiotic susceptibility assay allows us to determine the number of *C*. *suis* inclusions at different concentrations of tetracycline *in vitro*, it is a more precise parameter to evaluate the actual biological behavior of chlamydial isolates in the presence of antibiotics. Excluding all isolates with an intermediate phenotype, the positive predictive value of the *tet*(C) PCR is 81.5% compared to the phenotype and only 68.8% if the intermediate phenotype is included. In contrast, the negative predictive value of the *tet*(C) PCR was 100% indicating that all phenotypically resistant *C*. *suis* isolates carry the *tet*(C) gene (Fig 4).

### *Tet*(C) PCR performed directly from clinical swabs strongly increases the number of false negative results

So far, we have been able to show that *tet*(C)-negative results translate into a sensitive or intermediate phenotype *in vitro*. However, we used chlamydial DNA extracted from the isolates for these tests. In a second step, we aimed to investigate whether *tet*(C) PCR from clinical swabs yielded similar results.

**Fig 5:**
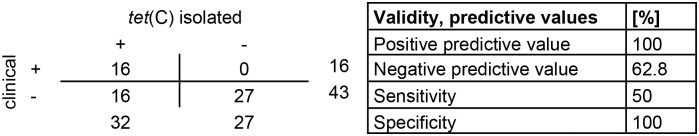
Presence of *tet*(C) in clinical swabs in comparison to their corresponding isolated samples. The left panel compares the *tet*(C) PCR values of the corresponding clinical sample (y-axis) with that of the isolate (x-axis) for each individual sample. The right panel displays the validity and predictive values for this comparison.

Interestingly, out of 32 isolated samples positive for *tet*(C), only 16 corresponding clinical swabs were positive resulting in a sensitivity of only 50% and a negative predictive value of 62.8% for clinical swabs. In contrast, all the *tet*(C) PCR negative isolates were also negative in the corresponding clinical samples (Fig 5).

Taken together, clinical swab samples are more frequently negative for *tet*(C) (n = 43) compared to isolated samples (n = 27), resulting in a greater number of false negative results. Furthermore, it must be noted that out of the 10 positive *tet*(C) results from either phenotypically sensitive or intermediate isolates mentioned in Fig 3, six were also positive in the clinical swab. Therefore, PCR analysis of clinical samples does not necessarily reduce the number of false positives.

### Isolates from pigs without antibiotic treatment were never phenotypically resistant

In Switzerland, prophylactic and metaphylactic oral antimicrobial treatment of fattening pigs is common [9]. In particular, tetracycline and tetracycline derivatives, as broad-spectrum antibiotics, have been used extensively in the pig industry for both prophylactic and therapeutic treatment [20]. As expected from previous publications [13], we found that farms treating pigs with oral tetracycline derivatives (CTS, Treatment Group C) harbored the most phenotypically resistant *C*. *suis* cultures with 18 out of 26 isolated samples (64.3%) (S2 Table). Of the remaining samples, six isolates were sensitive and two were intermediate. Farms without oral antimicrobial treatment during the fattening period (Treatment Group A) did not harbor any resistant isolates. Of 18 investigated cultures, 16 were sensitive and two belonged to the intermediate phenotype (S2 Table). Interestingly, from farms that treated their animals with trimethoprime, sulfadimidin and sulfathiazole (TSS, Treatment Group B), a highly diverse population of different *Chlamydia*-positive cultures was isolated. Out of 15 isolates tested *in vitro*, four were phenotypically resistant, nine were sensitive and two intermediate (S2 Table).

### Oral treatment with tetracycline during the fattening period increases the incidence of resistance in pig farms

**Fig 6:**
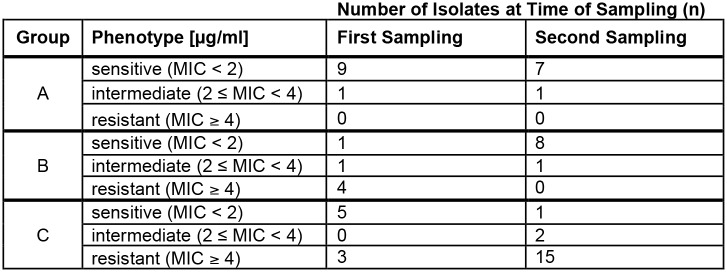
*In vitro* phenotypes within individual treatment groups at the first and second sampling. Shown is the phenotype *in vitro* (sensitive, intermediate, resistant) of each investigated isolate as estimated by antibiotic susceptibility assay under consideration of the sampling timepoint (first sampling at the beginning, second sampling at the end of the fattening period) and which treatment group the sampled pig belonged to: A (no antibiotics), B (TSS) or C (CTS).

Pigs were sampled both at the beginning (first sampling) and end (second sampling) of the fattening period [9]. We compared the *in vitro* phenotype of isolates obtained at the first with isolates obtained at the second sampling timepoint under consideration of the three treatment groups. For Treatment Group A (no antibiotics), the tetracycline susceptibility of 18 isolates originating from eleven pigs was determined, which resulted in nine phenotypically sensitive and one intermediate isolates at the first as well as seven sensitive and one intermediate phenotype at the second sampling. Interestingly, the four phenotypically resistant isolates originating from pigs in Group B (TSS) were altogether obtained at the first sampling. At the end of the fattening period, only the sensitive (n = 8) or intermediate (n = 1) phenotype was present. Within Group C (CTS), there was an increase of tetracycline resistant and a decrease of phenotypically sensitive isolates between the first and second sampling. In detail, only three out of eight investigated pigs harbored phenotypically resistant isolates (37.5%) at the first sampling while 15 out of 18 isolates were resistant in the antibiotic susceptibility assay at the second collection (83.3%). Furthermore, whereas five tetracycline sensitive isolates were obtained at the first sampling, only one was present at the second sampling. Additionally, two intermediate isolates were collected at the second sampling (Fig 6).

To further confirm these findings, individual pigs were selected of which successful isolation was performed both at the first and second sample collection resulting in 36 paired isolates originating from 18 pigs. In Group A (seven pigs), sensitive (n = 6) and intermediate (n = 1) phenotypes were present at the first sampling while only sensitive isolates remained at the second sampling. Individual pigs within Group B (six pigs) confirmed the previous observation that isolates could be phenotypically resistant at the first collection but cultures obtained from the same pig were sensitive at the second sampling (n = 4). Within Group C (five pigs), three out of five pigs harbored resistant isolates at both collection timepoints, whereas two pigs contained phenotypically sensitive isolates at the first and the intermediate or resistant phenotype at the second sampling (S3 Table).

## Discussion

Growing numbers of antibiotic resistant pathogens have caused the Swiss Federal Office of Public Health to specifically target and monitor the consumption of antibiotics in human and veterinary medicine [21]. This strategy was developed in addition to the total ban on the use of antibiotic growth promoters in livestock initialized by Sweden in 1986, implemented by Switzerland in 1999 and followed by the European Union (EU) in 2004 [22, 23].

The first tetracycline resistant *C*. *suis* strains were reported in USA pig farms in the early 1990s [7]. The relatively inexpensive tetracycline and tetracycline derivatives with broad spectrum activity are the most commonly used antibiotics with more than 6500 tons approved for domestic use in food-producing farm animals in 2014 in the USA [24].

In the current study, we aimed to compare the tetracycline susceptibility *in vitro* of porcine clinical samples with the presence or absence of the *tet*(C) gene, which required isolation of various *C*. *suis* samples previously identified by real-time PCR and subsequent species identification by Arraymate microarray [9]. We found that two factors mainly influenced the success of our isolation attempts: the bacterial load as estimated by quantitative real-time PCR, and the sampled anatomical site (rectal versus conjunctival).

Petayaev et al. [25] showed that as few as 200 copies per ml by quantitative TaqMan PCR were enough to successfully isolate *C*. *pneumoniae* from the sera of patients with acute coronary syndrome. In contrast to these findings, fewer than 1000 copies per ml (< 1 copy per μl) resulted in a success rate of only 25% in our study. However, it must be taken into consideration that, with *C*. *pneumoniae* and *C*. *suis*, two different chlamydial species were chosen for isolation and that quantification was performed with two different primer pairs, specifically a *C*. *pneumoniae*-specific and a more general *Chlamydiaceae* primer pair targeting a 194 bp fragment of the 16S rRNA and a 111 bp fragment of the 23S rRNA gene, respectively [18, 25]. Sampling inconsistencies such as insufficient insertion of the flocked swabs used for PCR analysis into the rectum/conjunctival sac or subsequent failure of the DNA extraction could explain these findings. Sampling inconsistencies could also explain the five samples with over 700 copies per μl that could not be isolated. Taken together, our findings confirm previous publications showing that, while quantitative PCR is highly sensitive, it does not differentiate viable from non-viable pathogens. In *Mycobacteria*, for example, a close linear correlation between quantitative real-time PCR and the conventional colony counting method has been demonstrated, showing that the colony counts are consistently below real-time PCR estimates [26].

As estimated by Hoffmann et al. [9], the mean number of *Chlamydia* 23S rRNA gene copies per μl is significantly lower for conjunctival swabs compared to fecal swabs. Different bacterial loads depending on the anatomical site and nature of sampling has also been demonstrated in a recent review systematically analyzing 29 publications on human genital *Chlamydia* infection involving over 40,000 participants where rectal swabs yielded the highest load for male patients contrasting females where cervical swabs clearly resulted in the highest bacterial load [27]. However, the low bacterial load alone does not explain the differences in the success rates of isolating from conjunctival or fecal swabs: out of the 15 swabs with fewer than 1 copy per μl, all four successfully obtained isolates originated from fecal swabs. These findings confirm previous results showing no correlation between the presence of conjunctivitis and chlamydial positivity [9]. In contrast, successful isolation of conjunctival swabs originally collected from asymptomatic pigs with only a history of conjunctivitis but not active infection has been demonstrated in another study [11]. Therefore, given the low bacterial load of our samples and that isolation from conjunctival swabs has succeeded before, we suggest that fecal contamination rather than active infection of the conjunctiva with viable bacteria was present in the pigs sampled in this study.

*C*. *suis* isolates were tested for their antibiotic susceptibility *in vitro* in order to identify the phenotype (sensitive, intermediate, resistant) as well as the presence or absence of the *tet*(C) gene by PCR. For most bacteria, antimicrobial susceptibility testing *in vitro* has been the gold standard to detect potential antibiotic resistance, although PCR-based techniques have been established in recent years. However, one of the primary constraints of this detection method is that the presence of a resistance gene does not automatically translate into resistant phenotypes *in vitro* and that alternative resistance mechanisms may be present [28]. Interestingly, only one form of tetracycline resistance in *C*. *suis* has been reported to date. Furthermore, the *in vitro* susceptibility test of *Chlamydia* to antimicrobials is not affordable for all laboratories as it is a time-consuming assay. Consequently, *tet*(C) PCR is more commonly used for detection than the antibiotic susceptibility assay [15, 29]. In the current study, we found clear evidence for the benefit of isolating samples prior to molecular analysis over *tet*(C) PCR directly from clinical swabs as well as the superiority of the tetracycline susceptibility assay with and without PCR analysis over *tet*(C) detection alone. First, while all phenotypically resistant isolates were positive for *tet*(C), there were a substantial number of false positive *tet*(C) results for phenotypically sensitive or intermediate isolates. These findings indicate that the presence of the *tet*(C) gene in *C*. *suis* is not necessarily associated with tetracycline resistant phenotype *in vitro* confirming findings of a recent publication from Italy [11]. False positive *tet*(C) results may have been caused by confounding genetic factors or the presence of other tetracycline resistant bacteria belonging to the gut microbiota [4, 5]. Moreover, co-infection with multiple strains originating from the same species has been reported in other *Chlamydia* species [30, 31]. Although isolation and cultivation is known to alter and select specific strains [30, 32], isolation does not guarantee the presence of clonally pure *C*. *suis* strains. It is therefore possible that mixed infections of sensitive and resistant strains occurred. Clonal purification and subsequent whole-genome sequencing for confirmation is necessary to ensure the complete absence of mixed infection. Second, the antibiotic susceptibility assay allows a tripartite classification system identifying sensitive, intermediate and resistant isolates as commonly implemented in routinely applied susceptibility profiles for extracellular bacteria [33] leading to a more nuanced classification as opposed to a positive or negative result for the presence of *tet*(C). The importance of implementing a tripartite classification is particularly obvious if we consider that the rate of false positive *tet*(C) results is considerably higher for the intermediate (2 μg/ml ≤ MIC < 4 μg/ml) than the sensitive (MIC < 2 μg/ml) phenotype. Third, we compared *tet*(C) results of DNA extracted from the isolates with their corresponding clinical swab samples to evaluate the test accuracy of *tet*(C) of uncultured clinical samples because it would be a time-saving alternative to isolation of samples in cell culture. We found that the frequency of false negative results in the clinical swab samples was very high, possibly due to low bacterial loads before isolation or PCR inhibitors within the clinical sample. In conclusion, PCR analysis of clinical swabs alone is not suitable to evaluate the prevalence of tetracycline resistant *C*. *suis* strains in a herd.

We investigated the effect of antibiotic treatment on the susceptibility of isolates originally sampled both at the beginning and end of the fattening period originating from pigs belonging to treatment groups A (no antibiotics), B (TSS) or C (CTS). As expected, the distribution of phenotypically resistant and sensitive isolates depended on the treatment groups. However, even under consideration of selective pressure [34], it is still striking that untreated pigs harbored strains exhibiting only the sensitive or intermediate phenotype as opposed to pigs treated with chlortetracycline, where the majority of tetracycline resistant samples were isolated both at the beginning and especially at the end of the fattening period. These findings offer clear evidence for the impact of selective pressure in terms of tetracycline resistance in *C*. *suis* and confirm previous results of a small-scale study where conjunctival swabs were taken both at the beginning and end of the fattening period. The swabs originated from randomly selected pigs with signs of conjunctivitis and were tested for the presence or absence of *tet*(C) before and after tetracycline treatment resulting in a clear increase of *tet*(C) positive clinical samples between the first and second sampling [13].

Despite a great number of publications investigating tetracycline resistance in *C*. *suis*, only two studies have analyzed samples at two or more different points in time [12, 13]. This is the first study additionally investigating the same pig both before and after antibiotic treatment (paired sampling). Identification of individual pigs at both samplings was possible with the help of the official identification number of the pig. Paired samples from pigs treated with chlortetracycline confirmed the hypothesis of selective pressure as they harbored *tet*(C) either already at the first sampling or acquired it during the fattening period. Similarly, only the sensitive phenotype was found at the second sampling for Group A giving further indication that tetracycline treatment leads to the selection of tetracycline resistant *C*. *suis* strains.

One possible explanation for the occurrence of tetracycline resistant *C*. *suis* already at the beginning of the fattening period is that the pigs acquired these strains before they were brought into the farms, probably during the weaning period. Another important aspect is the fact that most farms buy their fattening pigs from various weaning farms. This increases the risk of cross-contamination through reported fecal-oral transmission [35] between pigs from different farms within the first days after entry. As a result, farrow-to-finish farms are known to have a lower risk of *Chlamydiaceae* infection [9] as well as acquisition or transfer of tetracycline resistance across the herd.

Unexpected among our findings, the majority of the paired samples in group B (TSS) changed their phenotype from resistant to sensitive after oral group treatment. All *Chlamydia* spp. are resistant to trimethoprim. However, *C*. *trachomatis* and *C*. *suis* are susceptible to sulfonamides, whereas *C*. *pneumoniae* and *C*. *psittaci* are resistant [36, 37]. There are several possibilities to explain the disappearance of resistant *C*. *suis* isolates in this treatment group. For example, the sulfonamides may have eradicated all active *C*. *suis* infections with subsequent reinfection of sensitive strains. Another hypothesis is predominance of sensitive strains in group B due to the lack of appropriate selective pressure during the fattening period as resistance is often associated with reduced bacterial fitness [34]. Sensitive *C*. *suis* strains may dominate in the absence of tetracycline.

In order to fully understand the interaction between the resistant and sensitive phenotype, the presence as well as expression of *tet*(C) and the true dynamics of *C*. *suis* infection during the fattening period, possible mixed infection and reinfection, further experimental studies and genomic analyses are necessary.

In summary, the current study demonstrates that two factors are important for successful isolation: the bacterial load and anatomical site. There was a positive correlation between the bacterial load and success of isolation, while fecal swabs were used more successfully for isolation compared to conjunctival swabs. We present further evidence that the presence of *tet*(C) does not automatically translate into a resistant phenotype and that the antibiotic susceptibility assay is more reliable than molecular methods to predict the biological behavior of isolates under antibiotic treatment. Additionally, we found evidence for the benefit of isolation over analysis directly from swab samples to reduce the number of false negative *tet*(C) results. Finally, we show that absence of tetracycline treatment prevents or at least massively reduces the incidence of tetracycline resistant isolates, while treatment promotes the presence of resistance. In contrast, while the combination of trimethoprime, sulfadimidin and sulfathiazole was unable to clear infections on herd-level, it clearly reduced the number of tetracycline resistant isolates.

## Supporting Information

S1 Table Identification of swabs used for isolation. (XLSX)

S2 Table Overview of isolates used for antibiotic susceptibility. (XLSX)

S3 Table Paired swabs used for antibiotic susceptibility and *tet*(C) analysis. (XLSX)

S1 Fig Representative *tet*(C) PCR image. Shown is a representative image of a gel run after amplification of the *tet*(C) gene by PCR. The ladder is on Lane 1 followed by the positive control (Lane 2), tetracycline-sensitive *C*. *suis* strain S45 (Lane 3), six samples from three individual pigs (Lanes 4–9; Lanes 7–9 are positive) and the negative control on Lane 10.
(TIF)

## Abbreviations

*C.*: *Chlamydia*
CTS (Group C): Chlortetracycline with or without tylosin and sulfadimidin (antibiotic treatment on herd level)
EMEM: Eagle’s minimum essential medium
FCS: Fetal calf serum
Group A: No antibiotic treatment on herd level
Group B (TSS): *see TSS (Group B)*
Group C (CTS): *see CTS (Group C)*
Inclusion-forming units per mililiter: IFU/ml
MIC: Minimum inhibitory concentration
MBC: Minimum bactericidal concentration
rRNA: ribosomal ribonucleic acid
SP transport medium: sucrose phosphate transport medium
Tet(C): Tetracycline resistance class C gene
TSS (Group B): Trimethoprime, sulfadimidin and sulfathiazole (antibiotic treatment on herd level)
