# Supplementary figures and images for: Selective Pressure Promotes Tetracycline Resistance of Chlamydia Suis in Fattening Pigs
Source: PLoS One. 2016 Nov 28;11(11):e0166917. doi: 10.1371/journal.pone.0166917 (PMC5125646; doi:10.1371/journal.pone.0166917)

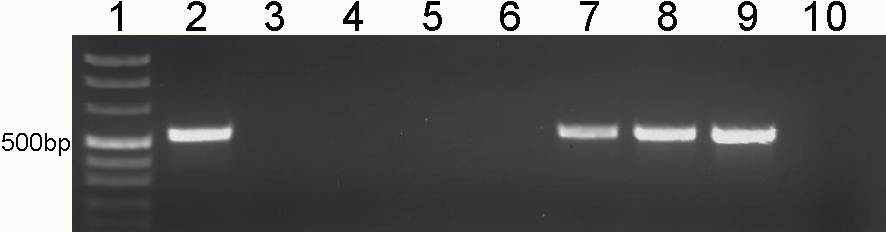

Supplement: S1 Fig — Shown is a representative image of a gel run after amplification of the tet(C) gene by PCR. The ladder is on Lane 1 followed by the positive control (Lane 2), tetracycline-sensitive C. suis strain S45 (Lane 3), six samples from three individual pigs (Lanes 4–9; Lanes 7–9 are positive) and the negative control on Lane 10. (TIF) [file pone.0166917.s004.tif]
